# Is robotic colorectal surgery worth the cost? An umbrella review integrating clinical outcomes, learning curve, and economic evidence

**DOI:** 10.1007/s11701-026-03716-8

**Published:** 2026-08-07

**Authors:** Silvia Martinelli, Alessio Lucarini, Paolo Aurello, Gianfranco Damiani, Niccolo Petrucciani

**Affiliations:** 1 Department of Life Sciences and Public Health, Università Cattolica del Sacro Cuore, Rome, Italy; 2 Clinique des Cedres, Ramsay Santé, Cornebarrieu, France; 3 Department of General Surgery and Organ Transplantation, San Camillo Forlanini Hospital, Rome, Italy; 4 Department of Medical and Surgical Sciences and Translational Medicine, St. Andrea Hospital, Sapienza University of Rome, via di Grottarossa 1035-9, Rome, 00189 Italy; 5 Department of Women, Child and Public Health Sciences, Fondazione Policlinico Universitario A. Gemelli IRCCS, Rome, Italy

**Keywords:** Robotic colorectal surgery, Umbrella review, Learning curve, Cost-effectiveness, AMSTAR 2, Conversion rate, Objective performance indicators

## Abstract

Robotic technology has progressively expanded within colorectal surgery owing to its enhanced instrument articulation and superior operative visualization. Despite these technical benefits, its adoption remains constrained by concerns regarding acquisition and procedural costs. Furthermore, the influence of surgeon proficiency and learning-curve progression on economic outcomes has rarely been considered in existing assessments of value. An umbrella review was conducted according to the Joanna Briggs Institute framework and reported in compliance with the PRISMA 2020 statement. A systematic search of PubMed, Embase, Scopus, and the Cochrane Library identified systematic reviews and meta-analyses published from January 2021 through March 2026 evaluating robotic versus laparoscopic colorectal procedures. Review quality was appraised using the AMSTAR 2 tool, while redundancy among included reviews was quantified through the Corrected Covered Area method. Twenty-seven eligible reviews encompassing more than 180 original studies and over 110,000 patients were analyzed. Across the available evidence, robotic colorectal surgery achieved perioperative and oncological outcomes comparable to those reported for laparoscopy, including rates of postoperative complications and anastomotic leakage. A consistent advantage was observed in the reduction of conversion to open surgery, particularly among patients undergoing rectal cancer resection and those with obesity. Although robotic procedures generally required longer operative times, this difference appeared closely linked to the initial phase of surgeon training. Economic analyses demonstrated greater upfront expenditures associated with robotic platforms; nevertheless, evidence from several methodologically robust reviews indicated that these disparities may narrow substantially once institutional and surgeon experience mature, especially in high-volume settings. Robotic colorectal surgery represents a reliable and effective minimally invasive approach that may provide specific technical benefits in challenging operative scenarios. Available cost-effectiveness studies may not fully reflect its long-term economic value, as improvements related to accumulated experience and learning-curve completion are seldom incorporated into current evaluation models.

**Supplementary Information:**

The online version contains supplementary material available at 10.1007/s11701-026-03716-8.

## Introduction

The development of robotic technology has expanded the armamentarium of minimally invasive colorectal surgery and has progressively gained acceptance in clinical practice. The robotic platform provides several technical features, including articulated instruments, stable three-dimensional imaging, and enhanced operator comfort, which may facilitate dissection and reconstruction during technically demanding procedures, particularly within the narrow confines of the pelvis. These characteristics have generated considerable interest in the potential of robotics to optimize surgical performance and improve perioperative outcomes.

However, whether these technical advantages translate into meaningful clinical benefits remains a subject of ongoing debate. Evidence from randomized controlled trials, systematic reviews, and meta-analyses has generally demonstrated similar results between robotic and laparoscopic colorectal surgery with regard to postoperative complications, mortality, and oncological quality indicators [1–3]. At the same time, robotic procedures are consistently associated with increased procedural and infrastructure-related costs. Consequently, the balance between clinical benefit and economic burden continues to represent a major issue when evaluating the role of robotic platforms in contemporary colorectal surgery [1–3].

Most economic evaluations comparing robotic and laparoscopic colorectal surgery rely on analyses performed at a single point in time and therefore provide only a partial representation of procedural value [3]. Such studies frequently neglect the progressive changes in surgeon performance that occur with accumulated experience. As a result, important determinants of cost and efficiency, including operative duration, conversion rates, postoperative outcomes, and resource consumption, may not be adequately captured. This issue is particularly relevant in robotic surgery, where proficiency develops gradually through a recognized learning process and performance metrics tend to improve as case volume increases [4].

Several investigations have reported that increasing experience with robotic platforms is associated with greater operative efficiency and improved perioperative outcomes, potentially reducing some of the excess costs observed during the initial adoption phase [2, 5]. Consequently, assessments restricted to early implementation periods may fail to reflect the long-term economic implications of robotic colorectal surgery. A more comprehensive evaluation should therefore consider how clinical performance evolves over time and how these changes influence resource utilization. Although a substantial amount of literature has explored robotic colorectal surgery, the available evidence remains dispersed among clinical, economic, and organizational perspectives. To date, these aspects have rarely been examined within a unified analytical framework. Moreover, no umbrella review has specifically evaluated the relationship between learning-curve progression and the interpretation of cost-effectiveness outcomes.

The present umbrella review was designed to synthesize evidence from systematic reviews and meta-analyses comparing robotic and laparoscopic colorectal surgery. Particular attention was devoted to perioperative and oncological outcomes, economic considerations, and the influence of surgeon experience on reported results. By integrating these complementary dimensions, this review aims to provide a broader understanding of the potential role and long-term value of robotic technology in modern colorectal surgery.

## Methods

### Study design

The present umbrella review was performed using the methodological framework proposed by the Joanna Briggs Institute (JBI) for conducting overviews of systematic reviews [6]. Reporting of the review adhered to the recommendations of the PRISMA 2020 statement [7], together with the specific guidance developed for overviews of reviews. Before study initiation, the review protocol was registered in the International Prospective Register of Systematic Reviews (PROSPERO; registration number CRD420251042541). The complete protocol can be consulted at https://www.crd.york.ac.uk/PROSPERO/view/CRD420251042541 (last accessed on June 9, 2026).

### Search strategy

A systematic search of the literature was undertaken across PubMed/MEDLINE, Embase, Scopus, and the Cochrane Library to identify relevant evidence published between January 2021 and March 2026. This time interval was selected to capture studies reflecting current-generation robotic systems, including the da Vinci Xi platform and subsequent technological developments, as well as contemporary perioperative management strategies. Search strategies incorporated both controlled vocabulary terms and text-word searches related to colorectal procedures, robotic and laparoscopic surgery, perioperative and oncological outcomes, surgeon learning and proficiency, and economic evaluation. The complete search strategy is reported in the Supplementary Material. In addition, the bibliographies of eligible reviews and abstracts from pertinent scientific meetings were screened to identify any additional relevant publications.

The following search string was used:

((“Colorectal Surgery“[MeSH] OR “colorectal surgery” OR “colon surgery” OR “rectal surgery” OR “colorectal neoplasms/surgery“[MeSH])

AND.

(“Robotic Surgical Procedures“[MeSH] OR robotic OR robot-assisted OR “robotic surgery”)

AND.

(“Laparoscopy“[MeSH] OR laparoscopic OR “minimally invasive surgery”)

AND.

(“Treatment Outcome“[MeSH] OR outcomes OR “clinical outcomes” OR morbidity OR mortality OR complications.

OR “Learning Curve“[MeSH] OR “learning curve” OR proficiency OR training.

OR “Costs and Cost Analysis“[MeSH] OR cost OR costs OR economic OR “cost-effectiveness”)

AND.

(“Systematic Review“[Publication Type] OR “Meta-Analysis“[Publication Type].

OR “systematic review” OR “meta-analysis” OR “umbrella review” OR “review of reviews”))

### Eligibility criteria

Studies were selected using the PICO framework:

*Population*: adult patients (> 18 years) undergoing colorectal surgery.

*Intervention*: robotic colorectal surgery.

*Comparison*: laparoscopic colorectal surgery.

*Outcomes*: clinical (morbidity, conversion, operative time, oncologic results), economic (costs, cost-effectiveness), and performance/learning-curve data.

*Inclusion*: systematic reviews and/or meta-analyses comparing robotic vs. laparoscopic colorectal surgery and reporting at least one of the above outcome domains; English language; human subjects. Exclusion: narrative reviews, editorials, letters, expert opinions; reviews without direct robotic-vs-laparoscopic comparison; reviews limited to highly specific techniques (e.g., lateral pelvic lymph-node dissection only); non-human studies; primary studies or conference abstracts.

### Study selection

**Fig. 1:**
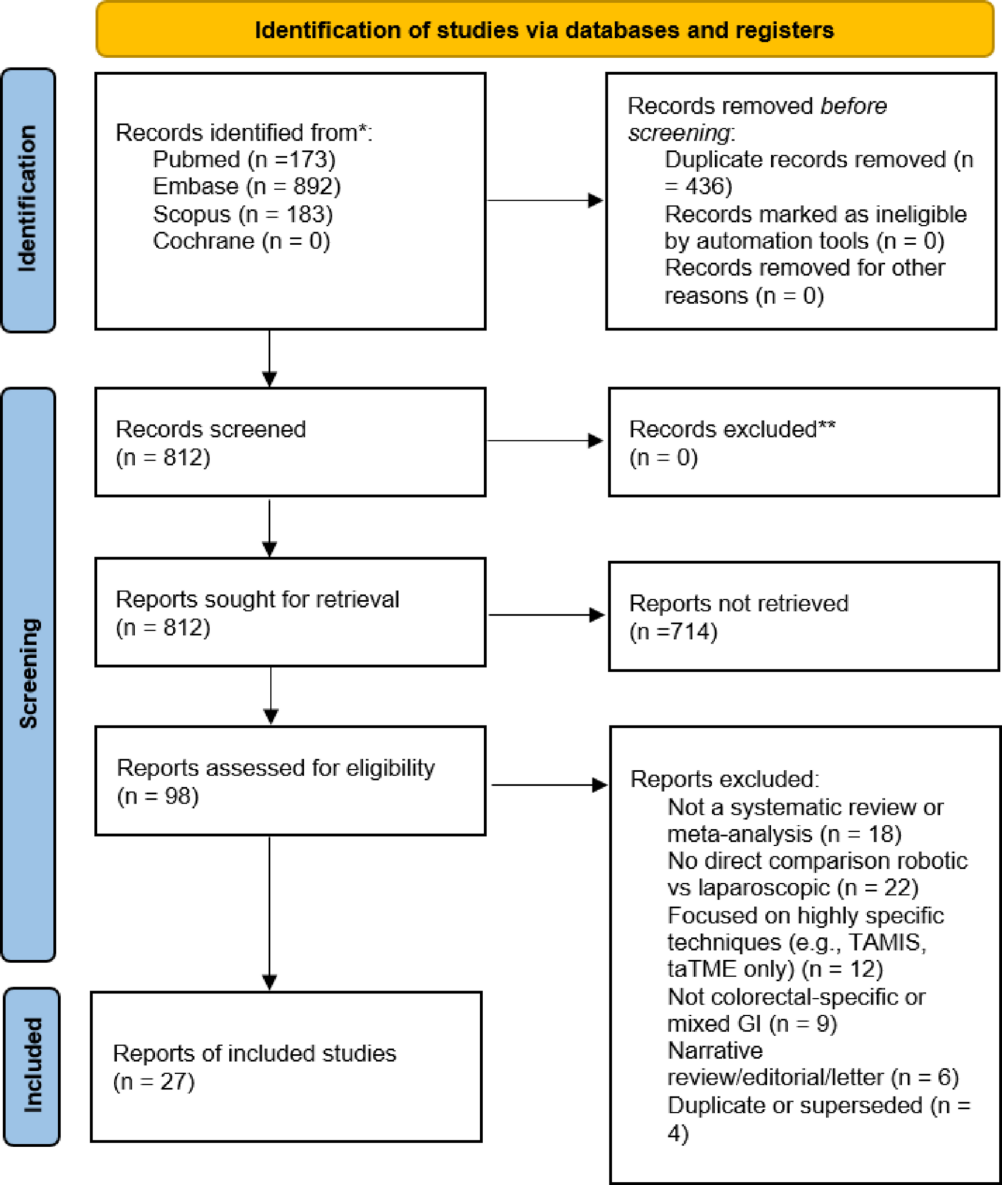
PRISMA 2020 flow diagram for new systematic reviews which included searches of databases and registers only. *Consider, if feasible to do so, reporting the number of records identified from each database or register searched (rather than the total number across all databases/registers). **If automation tools were used, indicate how many records were excluded by a human and how many were excluded by automation tools. Source: Page et al. [7]. This work is licensed under CC BY 4.0. To view a copy of this license, visit https://creativecommons.org/licenses/by/4.0/

Study selection was carried out independently by two reviewers through a two-stage process consisting of title and abstract screening followed by full-text assessment of potentially eligible articles. Any discrepancies were discussed and resolved through agreement between the reviewers. To evaluate the extent to which primary studies were represented in multiple reviews, overlap was assessed using the Corrected Covered Area (CCA) methodology [8]. This metric estimates the degree of redundancy among systematic reviews by considering both the total number of included reviews and the number of unique primary studies. According to established thresholds, CCA values of 0–5% were considered indicative of slight overlap, 6–10% of moderate overlap, 11–15% of high overlap, and values exceeding 15% of very high overlap. In instances of substantial overlap, greater weight was assigned to reviews with stronger methodological quality and more recent publication dates. Consequently, when multiple reviews addressed the same evidence base, interpretation of findings relied preferentially on the most up-to-date and methodologically robust sources in order to reduce the influence of duplicate evidence. The study selection process is summarised in the PRISMA flow chart (Fig. 1).

### Data extraction

Information from the eligible reviews was collected independently by two investigators using a predefined data extraction template. The extracted variables included bibliographic and methodological characteristics of each review, the design and number of primary studies, sample size, surgical procedures evaluated, reported clinical and economic outcomes, performance-related measures, and the extent to which learning-curve effects were considered in the analysis. To ensure accuracy and consistency, all extracted data underwent an additional verification process conducted by a third reviewer.

### Quality assessment

Methodological quality was evaluated using AMSTAR 2 [9], the recommended tool for assessing systematic reviews and meta-analyses. AMSTAR 2 includes 16 methodological domains and generates four overall confidence categories (High, Moderate, Low and Critically low) according to the presence of critical and non-critical weaknesses.

Methodological appraisal was conducted independently by two reviewers, with any differences in judgement resolved through discussion and agreement. Evidence synthesis was primarily informed by reviews classified as having high or moderate methodological quality, whereas findings from reviews rated as low or critically low quality were retained for completeness but interpreted with greater caution. The overall certainty of evidence for the principal outcomes was evaluated using the GRADE framework [10]. Given the umbrella-review design, GRADE assessments were applied at the level of the synthesized evidence, taking into account the methodological robustness of the included reviews, the consistency of findings across meta-analyses, and the degree of agreement among pooled effect estimates. Since GRADE was originally developed for primary evidence syntheses, certainty ratings were adapted for umbrella review methodology by integrating three complementary domains: (i) methodological quality of the included systematic reviews (AMSTAR 2), (ii) consistency of findings across independent meta-analyses, and (iii) overlap of the evidence base as assessed by the Corrected Covered Area.

### Data synthesis

Given the anticipated clinical and methodological heterogeneity among the included reviews, together with the presence of overlapping primary studies, results were synthesised narratively rather than through additional quantitative pooling. The evidence was organised into three predefined thematic areas: clinical outcomes, economic considerations, and measures related to surgeon performance and learning-curve progression. For each domain, particular attention was paid to the degree of agreement between reviews, potential explanations for heterogeneity, methodological weaknesses, and the corresponding GRADE assessments of evidence certainty. In light of the overlap between evidence sources and the objectives of the umbrella review, no de novo meta-analysis was undertaken.

## Results

### Study selection and characteristics

The database search identified 1248 potentially relevant records from PubMed/MEDLINE, Embase, the Cochrane Library, and Scopus. Following duplicate removal, 812 citations underwent title and abstract screening. Ninety-eight articles were subsequently reviewed in full text to determine eligibility (Fig. 1). After application of the predefined inclusion criteria, 27 systematic reviews and meta-analyses were retained for the final synthesis [2, 11–36] (Fig. 1). The characteristics of the included reviews are summarised in Table 1. The number of primary studies incorporated within individual reviews varied considerably, ranging from fewer than 10 to more than 20 studies, reflecting differences in review methodology, inclusion criteria, and publication periods. Collectively, the included reviews encompassed more than 180 unique primary investigations and over 110,000 patients (Table 1). The reviews were published between 2021 and 2026, with China representing the most frequent country of origin (*n* = 13). Most evidence focused on oncological colorectal resections, including right and left colectomies, sigmoid resections, and anterior rectal resections. Several reviews also explored specific patient subgroups, most commonly individuals undergoing rectal cancer surgery, patients with obesity, and elderly or otherwise high-risk populations.

**Table 1:** Characteristics of the 27 included systematic reviews and meta-analyses

Authors	Journal	Year	Country	Total number of studies included	Total number of patients
Filho et al.	Journal of Surgical Oncology	2025	Brazil	12	4856
Pompeu et al.	Journal of Laparoendoscopic & Advanced Surgical Techniques	2025	Portugal	15	7892
Zou et al.	BMC Surgery	2025	China	18	9214
Huang et al.	Frontiers in Oncology	2023	China	14	6783
Yang et al.	Clinical Research in Hepatology and Gastroenterology	2023	China	16	8451
Zhang et al.	International Journal of Surgery	2024	China	22	12,367
Tang et al.	World Journal of Surgical Oncology	2021	China	11	5672
Liu et al.	Journal of Robotic Surgery	2025	China	9	3214
Mirza et al.	Journal of Robotic Surgery	2025	UK	7	2856
Li et al.	Journal of Robotic Surgery	2025	China	13	6934
Abdelsamad et al.	Surgical Endoscopy	2024	Egypt	8	1987
Ishizuka et al.	European Journal of Surgical Oncology	2024	Japan	10	4567
Cao et al.	International Journal of Medical Robotics and Computer Assisted Surgery	2024	China	10	4123
Wu et al.	Computer Assisted Surgery	2026	China	11	5678
Li H et al.	World Journal of Surgical Oncology	2025	China	12	4912
McKechnie et al.	ANZ Journal of Surgery	2025	Canada	15	8456
Chen et al.	Journal of Robotic Surgery	2024	China	14	6789
Ammirati et al.	Minimally Invasive Therapy & Allied Technologies	2024	Italy	9	3,456
Wang et al.	Minimally Invasive Therapy & Allied Technologies	2025	China	10	4567
Gahunia et al.	Techniques in Coloproctology	2025	UK	8	2345
Rehman et al.	Cureus	2025	UK	12	5678
Flynn et al.	Colorectal Disease	2021	Australia	7	3214
Singh et al.	Translational Gastroenterology and Hepatology	2024	UK	13	16,082
Asmat et al.	Langenbecks Archives of Surgery	2024	Pakistan	11	4567
Liu et al. (2021)	Surgical Endoscopy	2021	China	14	7890
Abbass et al.	Disease of the Colon & Rectum	2022	Egypt	9	3456
Kowalewski et al.	Surgical Endoscopy	2021	Germany	8	2789

### Quality assessment

Methodological appraisal using the AMSTAR 2 instrument indicated an overall satisfactory level of quality among the 27 included systematic reviews and meta-analyses. Reviews based predominantly on randomized controlled trials generally achieved the highest ratings. Overall, 15 reviews (56%) were classified as High quality, 11 (41%) as Moderate quality, and 1 (4%) as Low quality, whereas no review met the criteria for a Critically low rating. Detailed results of the AMSTAR 2 assessment are presented in Table 2 and Supplementary Table S1. Despite the overlap of primary studies across reviews, the findings were remarkably consistent. Restricting the synthesis to reviews rated as High or Moderate quality did not materially alter the direction or magnitude of the reported effects for any major outcome, providing additional reassurance regarding the reliability of the overall evidence base. The most frequently encountered methodological limitations included the absence of prospective protocol registration (AMSTAR 2 Item 2), insufficient assessment of publication bias (Item 15, not adequately addressed in approximately 40% of reviews), and limited consideration of how risk of bias in primary studies might influence pooled results (Item 12). In contrast, higher-quality reviews, such as those by [13, 14, 27], were characterised by prospectively registered protocols, extensive literature searches, and more rigorous approaches to risk-of-bias evaluation.

**Table 2:** AMSTAR 2 domain-level assessment of the 27 included reviews. Critical domains (Items 2, 4, 7, 9, 11, 12, 15) are shown. Overall rating follows official AMSTAR 2 rules (Shea et al. BMJ 2017). High-quality reviews (*n* = 15) form the core of the synthesis

Study	1	2	3	4	5	6	7	8	9	10	11	12	13	14	15	16
Filho	Y	Y	Y	Y	Y	Y	PY	Y	Y	N	Y	Y	Y	Y	Y	Y
Pompeu	Y	Y	Y	Y	Y	Y	PY	Y	Y	N	Y	Y	Y	Y	Y	Y
Zou	Y	PY	Y	Y	Y	Y	N	Y	Y	N	Y	PY	Y	Y	Y	Y
Huang	Y	PY	Y	Y	Y	Y	N	Y	Y	N	Y	PY	Y	Y	Y	Y
Yang	Y	N	Y	Y	Y	PY	N	Y	PY	N	Y	PY	Y	Y	PY	Y
Zhang	Y	Y	Y	Y	Y	Y	PY	Y	Y	N	Y	Y	Y	Y	Y	Y
Tang	Y	Y	Y	Y	Y	Y	PY	Y	Y	N	Y	Y	Y	Y	Y	Y
Liu ‘25	Y	Y	Y	Y	Y	Y	PY	Y	Y	N	Y	Y	Y	Y	Y	Y
Mirza	Y	Y	Y	Y	Y	Y	PY	Y	Y	N	Y	Y	Y	Y	Y	Y
Li	Y	Y	Y	Y	Y	Y	PY	Y	Y	N	Y	Y	Y	Y	Y	Y
Abdelsamad	Y	PY	Y	Y	Y	PY	N	Y	PY	N	Y	PY	Y	Y	PY	Y
Ishizuka	Y	Y	Y	Y	Y	Y	PY	Y	Y	N	Y	Y	Y	Y	Y	Y
Cao	Y	PY	Y	Y	Y	PY	N	Y	PY	N	Y	PY	Y	Y	PY	Y
Wu	Y	Y	Y	Y	Y	Y	PY	Y	Y	N	Y	Y	Y	Y	Y	Y
Li H	Y	Y	Y	Y	Y	Y	PY	Y	Y	N	Y	Y	Y	Y	Y	Y
McKechnie	Y	Y	Y	Y	Y	Y	PY	Y	Y	N	Y	Y	Y	Y	Y	Y
Chen	Y	PY	Y	Y	Y	PY	N	Y	PY	N	Y	PY	Y	Y	PY	Y
Ammirati	Y	PY	Y	Y	Y	PY	N	Y	PY	N	Y	PY	Y	Y	PY	Y
Wang	Y	PY	Y	Y	Y	PY	N	Y	PY	N	Y	PY	Y	Y	PY	Y
Gahunia	Y	PY	Y	Y	Y	PY	N	Y	PY	N	Y	PY	Y	Y	PY	Y
Singh	Y	PY	Y	Y	Y	PY	N	Y	PY	N	Y	PY	Y	Y	PY	Y
Rehman	Y	PY	Y	Y	Y	PY	N	Y	PY	N	Y	PY	Y	Y	PY	Y
Flynn	Y	Y	Y	Y	Y	Y	PY	Y	Y	N	Y	Y	Y	Y	Y	Y
Asmat	Y	PY	Y	Y	Y	PY	N	Y	PY	N	Y	PY	Y	Y	PY	Y
Liu ‘21	Y	PY	Y	Y	Y	PY	N	Y	PY	N	Y	PY	Y	Y	PY	Y
Abbass	Y	PY	Y	Y	Y	PY	N	Y	PY	N	Y	PY	Y	Y	PY	Y
Kowalewski	Y	Y	Y	Y	Y	Y	PY	Y	Y	N	Y	Y	Y	Y	Y	Y

Legenda AMSTAR 2: Y = Yes; PY = Partial Yes; N = No

Importantly, the principal findings remained unchanged when greater emphasis was placed on the highest-quality evidence. Robotic colorectal surgery continued to demonstrate perioperative morbidity and oncological outcomes comparable to laparoscopy, together with a lower likelihood of conversion to open surgery, particularly in rectal cancer and obese populations. Longer operative times were consistently reported, although this difference appeared largely related to the early stages of robotic adoption and surgeon training. Economic evaluations continued to identify higher direct procedural costs for robotic surgery; however, reviews of higher methodological quality consistently suggested that cost disparities decrease substantially after surgeons achieve procedural proficiency, typically estimated at approximately 20–50 cases. These observations suggest that cost-effectiveness may improve in experienced high-volume centres; however, this hypothesis has not yet been consistently demonstrated in formal incremental cost-effectiveness analyses.

Furthermore, the limited incorporation of learning-curve effects into economic analyses remained evident even after restricting the assessment to higher-quality reviews. Evaluation of overlap using the Corrected Covered Area (CCA) revealed moderate-to-high redundancy of primary studies across several evidence domains (Table 3), including general colorectal cancer surgery (18%; very high overlap), rectal cancer surgery (22%; very high overlap), learning-curve studies (31%; very high overlap), and economic analyses (15%; high overlap).

**Table 3:** AMSTAR 2 Evaluation and CCA assessment of overlap among included systematic reviews

Domain	Num. of Reviews	AMSTAR 2 Evaluation	CCA Value (%)	Overlap Level
CRC General	8	4 High (Filho 2025, Pompeu 2025, Zhang 2024, Tang 2021) 3 Moderate (Zou 2025, Huang 2023, Yang 2023) 1 Low	18	Moderate
Rectal cancer (TME & subgroups)	7	5 High (Liu 2025, Mirza 2025, Li 2025, Ishizuka 2024, Kowalewski 2021) 2 Moderate (Abdelsamad 2024, Cao 2024)	22	Moderate-High
Obese patients	4	3 High (Wu 2026, Li H 2025, McKechnie 2025) 1 Moderate (Chen 2024)	12	Low-Moderate
Elderly/High-risk	3	3 Moderate (Ammirati 2024, Wang 2025, Gahunia 2025)	9	Low
Learning curve	2	1 High (Flynn 2021) 1 Moderate (Rehman 2025)	31	High
Economic/Cost analyses	1	1 Moderate (Singh 202)	15	Moderate
Specific outcomes (CRM, functional, complications)	2	1 High (Ishizuka 2024) 1 Moderate (Asmat 2024)	11	Low

Finally, the pre-specified sensitivity analysis including only the 26 reviews rated as High or Moderate quality produced results that were fully concordant with the primary analysis, confirming the robustness of the overall conclusions.

### Clinical outcomes

Across the available evidence, robotic and laparoscopic colorectal surgery achieved broadly comparable clinical results in most evaluated outcomes (Table 4). Rates of overall postoperative morbidity and major complications, defined as Clavien–Dindo grade III or higher, did not differ substantially between approaches. Some moderate-quality reviews reported a trend towards fewer complications in the robotic group; however, these differences generally failed to reach statistical significance. Similarly, the incidence of anastomotic leakage was comparable between the two techniques.

**Table 4:** Summary of clinical outcomes comparing robotic versus laparoscopic colorectal surgery across the included reviews

Outcome	Robotic vs. Laparoscopic	Consistency across reviews	AMSTAR 2-weighted interpretation	GRADE Quality
Overall complications	Similar (slight ↓ in robotic)	Moderate	No clear superiority	Moderate
Major complications (Clavien ≥ III)	Similar	Moderate-High	No significant difference	Moderate
Anastomotic leak	Similar	Moderate	No difference in high-quality reviews	Moderate
Conversion to open surgery	↓ Robotic (OR 0.27–0.57)	High	Consistent advantage, especially in rectal and obese patients	High
Operative time	↑ Robotic (mean + 25–50 min)	High	Largely attributable to early learning curve	High
Estimated blood loss	↓ Robotic	Moderate-High	Modest but consistent benefit	Moderate
Length of hospital stay	Similar	Moderate	Neutral	Moderate
Oncologic outcomes (R0, CRM, LN yield)	Equivalent	High	Non-inferiority confirmed in high-quality RCT-based reviews	High–Moderate
Functional outcomes (urinary/sexual/LARS)	Similar or slight benefit robotic	Moderate	High heterogeneity in measurement tools	Moderate–Low

The most consistent advantage associated with robotic surgery was a lower risk of conversion to an open procedure, with reported odds ratios ranging from 0.27 to 0.57. This benefit was particularly pronounced in technically challenging settings, including rectal cancer surgery and operations performed in obese patients. In contrast, robotic procedures were associated with longer operative durations, with reported mean increases ranging from approximately 25 to 50 min. Nevertheless, several high-quality reviews suggested that a substantial proportion of this difference reflects the initial learning phase associated with adoption of the robotic platform.

Robotic surgery was also associated with a modest reduction in intraoperative blood loss, although the magnitude of this benefit was generally limited. No meaningful differences were observed in hospital length of stay. From an oncological perspective, the two approaches demonstrated equivalent performance, including rates of R0 resection, circumferential resection margin involvement, and lymph-node retrieval.

Functional outcomes were less consistently reported. Available evidence suggested either comparable postoperative urinary, sexual, and bowel function outcomes or a slight advantage in favour of robotic surgery. However, interpretation of these findings is limited by substantial heterogeneity in outcome definitions and assessment instruments, particularly for low anterior resection syndrome (LARS).

According to the GRADE framework, the certainty of evidence was considered High for conversion rate, operative time, and oncological outcomes, whereas overall postoperative complications and anastomotic leak rates were supported by Moderate-certainty evidence (Table 5).

**Table 5:** GRADE assessment of the quality of evidence

Outcome	Main included reviews	AMSTAR 2 (review level)	No. of primary studies (RCT/Obs)	GRADE Quality of Evidence	Main reasons for rating/Notes
CRM positivity/R0 resection	Ishizuka 2024, Li 2025	High	4/2	High–Moderate	RCT predominant, low RoB, low heterogeneity
TME quality	Liu 2025, Tang 2021	High	3/0	High	Well-defined technical outcome, consistent results
Long-term OS/DFS	Zhang 2024, Pompeu 2025, Filho 2025	High	5/1	High	≥ 5-year follow-up in most studies
Conversion to open surgery	Wu 2026, Filho 2025, Zou 2025	High	5/3	High	Consistent effect across subgroups
Operative time	Zou 2025, Tang 2021	High	4/1	High	Consistently longer with robotic approach
Overall complications	Asmat 2024, Liu 2021	Moderate	2/4	Moderate	Mixed RoB, moderate heterogeneity
Anastomotic leak	Li 2025, Abdelsamad 2024	Moderate	3/2	Moderate	Low event rate, some imprecision
Urinary/sexual function	Mirza 2025, Abbass 2022, Kowalewski 2021	High–Moderate	2/3	Moderate–Low	Variable measurement tools, short follow-up
Functional outcomes (LARS)	Cao 2024, Mirza 2025	Moderate	2/1	Low	Subjective scores, high heterogeneity
Outcomes in obese patients	Wu 2026, Li H 2025, McKechnie 2025	High	4/2	High	Clear benefit in conversion and safety
Cost/cost-effectiveness	Singh 2024	Moderate	0/4	Low–Moderate	High heterogeneity between healthcare systems
Learning curve effects	Rehman 2025, Flynn 2021	Moderate	1/2	Moderate	Early-phase data, some variability

### Economic outcomes

Economic analyses consistently identified greater procedural expenditures for robotic colorectal surgery compared with laparoscopy (Table 6). The additional costs were primarily related to acquisition and maintenance of the robotic platform, use of dedicated disposable instruments and robotic stapling devices, and increased operating-room utilisation during the initial implementation phase. Instrument-related expenses represented a substantial component of the overall cost difference; however, several reviews reported that these expenditures could be reduced through optimisation of robotic instrument use, adoption of standardised procedural sets, and selective integration of conventional laparoscopic instruments when appropriate.

**Table 6:** Summary of economic outcomes comparing robotic versus laparoscopic colorectal surgery

Outcome	Finding	Consistency across reviews	Interpretation/Context	GRADE Quality
Direct costs	Significantly higher with robotic	High	Main driver: platform, instrumentation and longer operative time	Moderate
Operative room time cost	Higher in early phase	High	Strongly learning-curve dependent	Moderate
Cost per case (instrumentation)	Higher with robotic (especially SureForm/vessel sealer)	Moderate-High	Can be mitigated by standardized set + laparoscopic assistance	Low–Moderate
Cost-effectiveness	Mixed/context-dependent	Moderate	Not cost-effective in low-volume or early adoption; improves after learning curve	Low–Moderate
Long-term cost impact	Potential mitigation after proficiency	Moderate	High-volume centres: reduction of €360–1080 per case (console time) + ~€1362 with standardized instrumentation	Moderate

The economic attractiveness of robotic surgery varied considerably according to institutional experience and case volume. In centres with limited robotic activity or during the early stages of programme development, cost-effectiveness was generally unfavourable. Conversely, studies evaluating mature robotic programmes suggested a progressive reduction in procedure-related costs as surgeon proficiency increased. In particular, shorter console times achieved after completion of the learning curve were associated with estimated savings ranging from €360 to €1080 per procedure. Additional reductions of up to €1362 per case were reported when standardised instrumentation strategies were implemented. Interpretation of economic findings should be undertaken with caution, as substantial variability exists among healthcare systems, reimbursement models, and cost-accounting methodologies. Most available economic evidence derives from observational studies employing heterogeneous costing methodologies across different healthcare systems. Consequently, direct comparisons between studies should be interpreted cautiously.

Accordingly, the certainty of evidence for economic outcomes was graded as Low to Moderate (Table 5).

### Learning curve and performance

Consideration of learning-curve effects was relatively uncommon among the included reviews. Only 7 of the 27 reviews (approximately 26%) explicitly evaluated the influence of surgeon experience on clinical or economic outcomes (Table 7). Among these studies, the number of procedures required to achieve proficiency generally ranged from 20 to 50 cases, although the exact threshold varied according to operative complexity and the surgeon’s previous experience with minimally invasive colorectal surgery.

**Table 7:** Learning curve integration

Study	Learning curve addressed	Proficiency threshold	Effect on clinical outcomes	Effect on costs/efficiency	Notes
Flynn et al. (2021)	Yes	20–50 cases	Reduced operative time and conversion rate after threshold	Not assessed	Benchmark systematic review
Rehman et al. (2025)	Yes	Early phase focus	Comparable safety during early learning; longer operative time	Increased costs in early phase	Focused on initial adoption phase
Overall synthesis	Limited (7/27 reviews)	20–50 cases	Marked improvement after proficiency	Potential cost mitigation in high-volume centres	Major gap in economic evaluations

Across reviews addressing this topic, increasing experience with the robotic platform was consistently associated with progressive improvements in operative efficiency. Reductions in operative duration and conversion to open surgery were frequently observed after the proficiency phase had been reached, while maintaining comparable safety profiles and oncological standards. These findings suggest that performance metrics reported during the early adoption period may not accurately reflect the outcomes achievable after consolidation of technical expertise.

Despite the potential relevance of these observations, most economic analyses did not incorporate changes in performance over time and instead evaluated costs as static measures. Consequently, the impact of learning-related gains on long-term cost-effectiveness remains insufficiently explored in the current literature.

Assessment of overlap revealed the highest Corrected Covered Area (CCA) within the learning-curve domain, reaching 31% (Table 3). To limit the influence of duplicate primary studies, greater emphasis was placed on the most recent reviews with high methodological quality, particularly those reported in references [2, 34].

## Discussion

By integrating evidence from 27 contemporary systematic reviews and meta-analyses, this overview provides a broad assessment of the role of robotic technology in colorectal surgery. Overall, the available literature suggests that both approaches achieve similar levels of safety and oncological effectiveness. Rates of postoperative morbidity, major complications, anastomotic leakage, hospital stay, and pathological quality indicators, including R0 resection rates, circumferential margin status, and lymph-node harvest, were largely equivalent across studies (Table 4). These findings are in agreement with the conclusions of recent high-quality evidence syntheses, including those reported by Mirza et al. [14] and Filho et al. [12], which failed to demonstrate a clear clinical advantage of one minimally invasive approach over the other, even in technically demanding settings.

Among the outcomes synthesized by the included systematic reviews and meta-analyses, the reduction in conversion to open surgery emerged as the most consistent advantage associated with robotic technology. This observation was reproduced across multiple reviews with high methodological quality and remained evident in both randomized and observational evidence syntheses [11, 20]. Reported effect sizes were clinically meaningful, particularly in rectal cancer surgery and in patients with obesity, two scenarios in which limited operative space and difficult exposure may increase procedural complexity. The biological rationale supporting this finding is coherent with the technical characteristics of robotic systems, including enhanced instrument articulation, stable three-dimensional visualisation, and improved manoeuvrability within confined anatomical spaces [1, 37]. The reproducibility of this result across different patient populations and study designs reinforces confidence in its validity.

In contrast, robotic procedures were generally associated with longer operative durations. This difference, reported in most included reviews, ranged from approximately 25 to 50 additional minutes compared with laparoscopy. However, the interpretation of operative time requires consideration of surgeon experience. Reviews specifically evaluating proficiency development consistently showed that operative efficiency improves substantially with increasing case volume [2, 34]. Importantly, operative duration is multifactorial and may also be influenced by case complexity, patient selection, institutional workflow, the robotic platform used, operating-room team experience, and teaching activities.

After completion of the learning phase, operative times progressively decrease while maintaining equivalent safety and oncological standards. Evidence derived from CUSUM analyses further supports this concept, demonstrating identifiable proficiency thresholds, commonly reached after approximately 16–30 procedures depending on case complexity and surgeon background [38]. Broader literature reviews have reported wider ranges, extending from approximately 12 to more than 70 cases, reflecting differences in training environments, institutional support, and previous laparoscopic expertise [39].

Increasing familiarity with the robotic platform has been associated with improvements in workflow efficiency, optimisation of resource utilisation, and enhanced perioperative outcomes [5]. These observations suggest that outcomes measured during the early implementation period may not accurately represent the performance achievable after consolidation of technical expertise.

The economic findings of this umbrella review should therefore be interpreted within this dynamic context. Consistent with previous reports, robotic surgery was associated with higher direct procedural costs than laparoscopy [3, 27]. Capital acquisition, maintenance contracts, dedicated robotic instrumentation, and longer operating-room utilisation were identified as the principal contributors to these differences. Furthermore, substantial variability was observed among economic studies, with results strongly influenced by institutional volume, reimbursement models, and local healthcare structures (Table 6).

Notably, studies that incorporated the effects of surgeon experience and procedural maturation reported a progressive reduction in cost differentials over time. Improvements in operative efficiency and workflow organisation translated into measurable reductions in resource consumption, supporting findings from time-driven activity-based costing analyses [40, 41]. These data suggest that traditional cross-sectional cost comparisons may provide an incomplete representation of the long-term economic impact of robotic surgery.

From a health-economic perspective, the evaluation of robotic colorectal surgery should extend beyond simple comparisons of procedural expenditure. Contemporary health technology assessment frameworks emphasise the importance of considering incremental costs alongside clinical benefits and downstream consequences throughout the patient pathway [42, 43]. In this context, factors such as lower conversion rates, potential reductions in complication-related resource consumption, and progressive gains in operative efficiency may contribute to the overall value generated by robotic platforms. Nevertheless, robust incremental cost-effectiveness analyses remain relatively uncommon, and available estimates are highly dependent on local organisational characteristics, procedural volume, and the stage of programme development, limiting the transferability of results between healthcare systems [27].

Despite these observations, fewer than one-third of the included reviews explicitly considered surgeon experience when interpreting outcomes, and only a minority incorporated learning-related variables into economic analyses. This omission is noteworthy because robotic surgery follows a relatively structured process of skill acquisition, facilitated by features such as ergonomic console design, motion scaling, and tremor filtration [34]. The evidence synthesised in the present review, together with available CUSUM-based studies, indicates that performance evolves predictably with experience. Consequently, evaluations performed during the implementation phase may overestimate costs while underrepresenting the efficiency and stability achievable after proficiency has been attained.

### Clinical and organisational implications

The findings of this umbrella review support the use of robotic surgery as a valid minimally invasive option for colorectal procedures. Although overall clinical outcomes are largely comparable to laparoscopy, robotics may offer practical advantages in situations characterised by increased technical complexity, including rectal cancer surgery, obesity, narrow pelvic anatomy, and post-neoadjuvant dissection planes. In these settings, the reduction in conversion rates may represent a meaningful clinical benefit.

At an organisational level, the results highlight the importance of structured implementation strategies. Concentration of robotic activity within high-volume centres, formal proctorship programmes, dual-console mentoring, and systematic performance monitoring may facilitate earlier attainment of proficiency and reduce variability among surgeons. Consequently, successful adoption of robotic colorectal surgery requires not only investment in technology but also development of dedicated educational pathways and objective performance-assessment systems, including tools such as Intuitive Hub metrics and risk-adjusted CUSUM monitoring.

### Limitations

Several limitations should be considered when interpreting the findings of this review. As with all umbrella reviews, the conclusions depend on the quality and scope of the available systematic reviews and meta-analyses. Although methodological quality was generally favourable, as demonstrated by the AMSTAR 2 assessment (56% High and 41% Moderate quality reviews), some methodological weaknesses persisted, particularly regarding protocol registration and assessment of publication bias.

Another potential source of bias relates to overlap among primary studies. Corrected Covered Area values ranged from 9% to 31% depending on the outcome domain, indicating moderate-to-high redundancy in certain areas, particularly rectal cancer and learning-curve analyses. However, prioritisation of the most recent and methodologically robust reviews was adopted to minimise the influence of duplicate evidence.

The interpretation of economic outcomes is further complicated by substantial heterogeneity in costing methodologies, reimbursement systems, and healthcare structures across countries. Although several studies suggest that increasing surgeon experience may improve the economic performance of robotic surgery, robust evidence based on prospective incremental cost-effectiveness analyses remains limited. Therefore, these findings should be regarded as hypothesis-generating rather than definitive.

In addition, the decision to focus on reviews published from 2021 onwards was intended to ensure relevance to contemporary robotic technology, although this approach may have excluded some earlier evidence. Older studies were therefore considered selectively within the discussion when judged informative for contextual interpretation.

Although we addressed study overlap by prioritizing the most recent and methodologically robust systematic reviews, high CCA values indicate that several conclusions rely partly on overlapping primary datasets. Consequently, the apparent consistency across systematic reviews should not be interpreted as fully independent confirmation of the evidence, and the certainty of the findings should be interpreted accordingly.

It should be acknowledged that, by design, umbrella reviews synthesize evidence derived from systematic reviews and meta-analyses rather than individual primary studies. Nevertheless, recently published randomized controlled trials deserve consideration when interpreting the current evidence. In particular, the REAL trial reported improved oncological outcomes with robotic colorectal surgery compared with laparoscopy, while the pilot RoLaCaRT randomized trial also suggested a trend toward improved oncological outcomes in the robotic group. Although these studies were not eligible for inclusion in the present umbrella review because they are primary investigations rather than evidence syntheses, they provide important contemporary evidence that supports the need for future updated systematic reviews and meta-analyses to reassess the oncological impact of robotic colorectal surgery [44, 45].

Finally, long-term evidence remains limited. Few reviews reported outcomes extending beyond five years, particularly with regard to oncological durability, functional recovery, and patient-reported quality-of-life measures. Additional research will be required as more mature follow-up data become available.

### Strengths

This review also possesses several strengths. Methodological transparency was enhanced through prospective registration in PROSPERO, adherence to PRISMA and PRISMA-for-Overviews recommendations, independent study selection and data extraction, systematic AMSTAR 2 appraisal, and formal evaluation of review overlap using the CCA methodology (Table 3). Furthermore, unlike many previous evidence syntheses, the present review specifically examined the relationship between learning-curve progression and economic evaluation, thereby addressing a dimension that has often received limited attention in the colorectal robotic literature. The incorporation of GRADE assessments (Table 5) additionally provides a transparent framework for interpreting the certainty of evidence across outcomes.

#### Future research directions

Future investigations should move beyond static comparisons of robotic and laparoscopic surgery and instead adopt models capable of capturing changes in performance over time. Integration of risk-adjusted CUSUM analyses, objective performance indicators, and proficiency milestones into economic evaluations would provide a more realistic representation of the value generated by robotic programmes. Prospective multicentre registries collecting standardised information on console utilisation, instrument consumption, operative efficiency, and patient-reported outcomes would be particularly valuable. In addition, comparative evaluations of emerging robotic platforms and newer system generations, including comparisons between da Vinci Xi and da Vinci 5 technologies, may help clarify the most effective strategies for implementation and training.

The rapid emergence of new robotic platforms from several manufacturers is likely to increase market competition, potentially reducing acquisition and maintenance costs while improving access to robotic technology. Future economic evaluations should therefore consider these evolving market dynamics, as findings based exclusively on earlier da Vinci generations may not remain applicable over time.

## Conclusions

The evidence synthesized in this umbrella review indicates that robotic colorectal surgery achieves clinical and oncological outcomes comparable to laparoscopy while offering specific technical advantages, particularly in complex surgical scenarios. Although robotic surgery remains associated with higher procedural costs, current economic evidence is largely based on heterogeneous observational studies and rarely incorporates changes in performance associated with increasing surgical experience. Consequently, the long-term cost-effectiveness of robotic colorectal surgery remains uncertain and should be evaluated through prospective economic studies integrating learning-curve progression, institutional maturity, and standardized cost-effectiveness methodologies.

## Electronic Supplementary Material

Below is the link to the electronic supplementary material.

Supplementary File 1

## Data Availability

No datasets were generated or analysed during the current study.
